# Risk of Deaths, AIDS-Defining and Non-AIDS Defining Events among Ghanaians on Long-Term Combination Antiretroviral Therapy

**DOI:** 10.1371/journal.pone.0111400

**Published:** 2014-10-23

**Authors:** Fred Stephen Sarfo, Maame Anima Sarfo, Betty Norman, Richard Phillips, George Bedu-Addo, David Chadwick

**Affiliations:** 1 Komfo Anokye Teaching Hospital, Kumasi, Ghana; 2 Kwame Nkrumah University of Science and Technology, Kumasi, Ghana; 3 The James Cook University Hospital, Middlesbrough, United Kingdom; Faculty of Medicine, Australia

## Abstract

Combination antiretroviral therapy (cART) has been widely available in Ghana since 2004. The aim of this cohort study was to assess the incidences of death, AIDS-defining events and non-AIDS defining events and associated risk factors amongst patients initiating cART in a large treatment centre. Clinical and laboratory data were extracted from clinic and hospital case notes for patients initiating cART between 2004 and 2010 and clinical events graded according to recognised definitions for AIDS, non-AIDS events (NADE) and death, with additional events not included in such definitions such as malaria also included. The cumulative incidence of events was calculated using Kaplan Meier analysis, and association of risk factors with events by Cox proportional hazards regression. Data were closed for analysis on 31^st^ December, 2011 after a median follow-up of 30 months (range, 0–90 months). Amongst 4,039 patients starting cART at a median CD4 count of 133 cells/mm^3^, there were 324 (8%) confirmed deaths, with an event rate of 28.83 (95% CI 25.78–32.15) deaths per 1000-person follow-up years; the commonest established causes were pulmonary TB and gastroenteritis. There were 681 AIDS-defining events (60.60 [56.14–65.33] per 1000 person years) with pulmonary TB and chronic diarrhoea being the most frequent causes. Forty-one NADEs were recorded (3.64 [2.61–4.95] per 1000 person years), of which hepatic and cardiovascular events were most common. Other common events recorded outside these definitions included malaria (746 events) and respiratory tract infections (666 events). Overall 24% of patients were lost-to-follow-up. Alongside expected risk factors, stavudine use was associated with AIDS [adjusted HR of 1.08 (0.90–1.30)] and death (adjusted HR of 1.60 [1.21–2.11]). Whilst frequency of AIDS and deaths in this cohort were similar to those described in other sub-Saharan African cohorts, rates of NADEs were lower and far exceeded by events such as malaria and respiratory tract infections.

## Introduction

Combination anti-retroviral therapy (cART) for the long-term management of HIV infection is administered to achieve long-term suppression of virological replication and to maintain CD4 cell counts at a level that reduces the risk of morbidity and mortality. It is encouraging that the effectiveness of cART in developing countries in sub-Saharan Africa has been reported to be similar, and often superior in clinical and immunologic outcomes when compared with those from the developed countries [1]–[8]. Evidence of the sustainability of these initially favourable immunological and clinical responses is beginning to emerge.

Deaths in the era of cART have largely been due to AIDS-defining clinical events in many such reports from developing countries. But the dynamics of mortality is believed to be changing in industrialised countries with non-AIDS defining clinical events assuming greater importance as causes of death as patients live longer on potent cART [9]–[11]. Non-AIDS defining events are classified as cardiovascular, renal, hepatic-related or non-AIDS-defining malignancies that are likely to have an impact on morbidity and mortality [12]. One report from Botswana indicated that the age-standardised incidence rates of non-AIDS defining events were comparable to those in the United States [13]. However, the spectra of disease entities included in this definition is debated [14], [15] and does not capture infectious diseases such as malaria which is a common cause of morbidity among patients in sub-Saharan Africa.

Ghana like many other countries in sub-Saharan Africa started cART roll-out in 2004. We have recently published a comparative analysis of the effectiveness and tolerability of nevirapine and efavirenz based cART among a large cohort of Ghanaian HIV-infected patients [16]. The aim of this study is to present a comprehensive analysis of the incidence, causes and risk factors associated with AIDS, non-AIDS clinical events, immunological failure, immune reconstitution inflammatory syndrome, treatment-limiting toxicity, and mortality over the long-term in this Ghanaian cohort.

## Methods

Ethical permission for this study was given by the Committee on Human Research Publications and Ethics of the Kwame Nkrumah University of Science and Technology and the Komfo Anokye Teaching Hospital, Kumasi, Ghana (ref: CHRPE/AP/073/13). Our institutional review board waived the need for a written informed consent since this was a retrospective, observational study and anonymised data were collected from patients' records. The study was conducted at the HIV clinic at the Komfo Anokye Teaching Hospital in Kumasi, Ghana, which provides HIV care to a large rural and urban population across central and northern Ghana. Antiretroviral therapy has been administered to patients meeting eligibility criteria since 2004 as has been previously described [16]. Data were extracted from the notes of patients starting ART between January 2004 and December 2010 and was closed for analysis by an intention-to-treat basis on 31^st^ December 2011. For this analysis, AIDS-defining events, non-AIDS defining clinical events, immune reconstitution inflammatory syndrome, loss-to-follow up, death and adherence to therapy were defined as follows. An AIDS-defining clinical event was defined as the occurrence of any opportunistic infections or malignancy according the World Health Organisation (WHO) [17] criteria while the patient was on cART.

The diagnosis of a non-AIDS clinical event was adjudicated by consensus between FSS, BN and RP (BN and RP are Consultants in HIV Medicine at the Komfo Anokye Teaching Hospital). The conditions classified under NADEs included cerebrovascular accident (stroke), cerebral/sub-arachnoid hemorrhage, myocardial infarction, coronary artery disease, congestive cardiac failure, end-stage renal disease, renal failure, cirrhosis of the liver, esophageal varices, hepatic failure, hepatic coma, hepatic encephalopathy, intestinal adenocarcinoma/lymphoma, penile carcinoma, small cell lung carcinoma, malignant melanoma, hepatocellular carcinoma, squamous cell carcinoma, and squamous cell carcinoma of the anus as previously described [14], [15]. Severity of specific NADEs in particular liver injury and renal failure were assessed using established Division of AIDS (DAIDS) tables for Grading Severity of Adult Adverse Experiences (NADEs).

Other medical diagnoses that did not fulfil the criteria for AIDS-defining events or serious NADEs were recorded and presented as “medical co-morbidities on cART”. These included conditions such as malaria, urinary tract infections, new onset diabetes mellitus or hypertension.

In the present study immune reconstitution inflammatory syndrome (IRIS) was defined as the paradoxical worsening of a previously treated opportunistic infection [18] for which the clinician was able to demonstrate radiological evidence of exacerbation in the cases of tuberculosis or cerebral toxoplasmosis, and fundoscopic evidence of vitreous inflammation in the case of CMV retinitis. Cases of herpes zoster IRIS were defined clinically. Due to the inherent difficulty of differentiating an opportunistic infection with normal presentation and a disorder with presentation that is compatible with unmasking IRIS in our setting, the unmasking type of IRIS was not documented in charts in this cohort. Immunological failure was defined using WHO criteria as either the return of CD4 counts to pre-therapy baseline, or below and/or more than 50% fall from on-therapy CD4 peak-level (and/or more than 50% fall in CD4), or persistent low CD4 of less than 100 cells/µl after one year of therapy without other concomitant infection to explain the low CD4. Loss to follow up was defined as missing a clinic appointment by at least 3 months of the last scheduled visit to clinic. Death was defined as the demise of a patient from causes related to HIV/AIDS or from toxicity from antiretrovirals or other non-AIDS related causes if known. Confirmation of death was by death certification by medical doctors for patients who died on the wards or by patients' family for those who died outside the hospital. Patient adherence, assessed by pill count, was classified as excellent at each clinic visit if adherence level of ≥95% was achieved. Poor adherence was defined as any documented evidence of <95% of adherence during follow up.

### Statistical analysis

Parametric and non-parametric methods were used to compare baseline characteristics of continuous data between patients started on either efavirenz, nevirapine or a protease inhibitor-based ART. A 1-way analysis of variance (ANOVA) or Kruskal-Wallis test was used to compare means or medians respectively. Comparisons of dichotomous data were performed using χ^2^ or Fisher's exact test. Crude incidence rates of events were calculated in person-years of follow-up with 95% confidence intervals calculated using Normal approximation to the Poisson distribution. Risk factors associated with death and AIDS-defining events were assessed using multivariable Cox proportional hazards regression with factors attaining a significance level of <0.10 in univariate analyses included in the final model. For these survival analyses, the month in which patients started cART was set as month zero and one day of follow-up was added to patients who did not attend any follow-up visits after initiating therapy. Time to events of interest, namely AIDS-defining events, loss-to-follow up or mortality, was calculated by subtracting the date of the event from the date on which the patient was started on cART. Patients were censored at December 31, 2011 if there were no event of interest. Explanatory variables included in survival analyses were selected on the basis of their well-recognised impact on clinical outcomes such as AIDS-defining events, loss-to-follow up and deaths. The cumulative incidence of loss to follow up and deaths were calculated using the Kaplan Meier methodology. For all analysis, a 2-sided p-value <0.05 was set as the level of statistical significance. All data analyses were conducted using SPSS version 19.

## Results

### Baseline demographics and laboratory characteristics of study participants

Four thousand and thirty-nine (4,039) patients out of 10,500 (38.5%) patients registered between January 2004 and December 2010 initiated first line therapy. There was a female preponderance in a ratio of 2.1: 1.0 with a median age of patients of 38 years (range of 14–78). As shown in Table 1, 2,376 (58.8%) patients were started on efavirenz based cART compared with 1,623 (40.2%) on nevirapine based cART while 40 (1.0%) were initiated on protease inhibitor based cART because they had HIV-2 mono-infection or HIV-1/2 dual infection: 23 on ritonavir-boosted lopinavir and 17 on nelfinavir. 52.1% were started on an NRTI backbone of stavudine (d4T) and lamivudine (3TC) while 47.7% were initiated on zidovudine (AZT) plus 3TC with 0.2% commencing other NRTIs.

**Table 1:** Baseline demographic, clinical and laboratory characteristics of patients initiating cART.

Characteristic	Efavirenz n = 2,376	Nevirapine n = 1,623	Protease inhibitors n = 40	Total n = 4,039	p-value
Male: female	1,028: 1,348	248: 1,375	11: 29	1,287: 2,752	<0.0001
Median (range) age	40 (14–77)	35 (15–75)	36 (25–65)	38 (14–77)	<0.0001
WHO clinical stage n (%)					0.09
1	165 (6.9)	106 (6.5)	2 (5.0)	273 (6.8)	
2	258 (10.9)	225 (13.9)	6 (15.0)	489 (12.1)	
3	1274 (53.6)	867 (53.4)	25 (62.5)	2166 (53.6)	
4	407 (17.1)	238 (14.7)	10 (10.0)	649 (16.1)	
No data	272 (11.4)	187 (11.5)	3 (7.5)	462 (11.4)	
Mean BMI ± SEM	20.1±0.09	20.5±0.10	21.0±0.62	20.3±0.07	0.0025
BMI categories					
<18.5 kg/m^2^	868 (36.5)	543 (33.5)	11 (27.5)	1422 (35.2)	0.0047
18.5–24.5 kg/m^2^	1145 (48.2)	804 (49.5)	20 (50.0)	1969 (48.8)	
>24.5 kg/m^2^	291 (12.3)	246 (15.2)	6 (15.0)	543 (13.4)	
No data	72 (3.0)	30 (1.8)	3 (7.5)	105 (2.6)	
CD4 count Median (range)	127.5 (1–1085)	140.0 (0–676)	186.0 (1–1134)	134 (0–1134)	0.0006
CD4 categories					
<200 cells/ml	1684 (70.9)	1080 (66.5)	21 (52.5)	2785 (69.0)	<0.0001
200–350 cells/ml	611 (25.7)	495 (30.5)	12 (30.0)	1118 (27.7)	
>350 cells/ml	53 (2.2)	41 (2.5)	7 (17.5)	101 (2.5)	
No data	28 (1.2)	7 (0.5)	0 (0.0)	35 (0.9)	
Median (range) Hemoglobin (g/dl)	10.2 (2.6–19.4)	10.2 (3.2–19.8)	10.9 (6.8–16.3)	10.2 (2.6–19.8)	0.07
Mean ± SEM ALT (U/L)	40.5±0.87	32.0±0.75	39.3±6.64	37.1±0.60	<0.0001
Mean ± SEM AST (U/L)	53.6±0.93	45.2±0.95	51.0±11.6	50.2±0.68	<0.0001
eGFR (ml/min/1.73m^2^) Median (IQR), n	64.0 (47.0–83.0) n = 1791	71.0 (54.0–89.0) n = 1240	66.5 (54.5–89.5) n = 32	66.0 (50.0–86.0) n = 3063	<0.0001
eGFR categories					
>60ml/min	1028 (57.4)	849 (68.5)	21 (65.6)	1898 (62.0)	<0.0001
30–59 ml/min	638 (35.6)	345 (27.8)	11 (34.4)	994 (32.5)	
15–29 ml/min	92 (5.1)	35 (2.8)	0 (0.0)	127 (4.1)	
<15ml/min	33 (1.8)	11 (0.9)	0 (0.0)	44 (1.4)	
HBV co-infection Positive/Negative (%)	143/761 15.8%	87/527 14.2%	3/13 18.8%	233/1301 15.2%	>0.05
NRTI backbone					
AZT +3TC	1083 (45.6)	819 (50.5)	23 (57.5)	1925 (47.7)	<0.0001
D4T +3TC	1286 (54.1)	804 (49.5)	13 (32.5)	2103 (52.1)	
Others	7 (0.3)		4 (10.0)	11 (0.2)	

BMI-Body Mass Index; ALT-Alanine transaminitis; AST- Aspartate transaminitis; eGFR- estimated glomerular filtration rate calculated using Cockroft Gault formula; AZT-zidovudine; 3TC- Lamivudine; d4T- stavudine.

**Table 2:** Enrolment, characteristics, follow-up and vital status of patients initiating cART according to calendar year of enrolment.

Year Characteristic	2004	2005	2006	2007	2008	2009	2010	TOTAL	p-value
No. enrolled for ART	1,700	2,020	1,819	1,782	1,738	636	705	10,400	
No. starting ART	769	695	819	658	590	272	236	4039	
% starting ART	45.2	34.4	45.0	36.9	33.9	42.8	33.5	38.8	
WHO Clinical stage, n (%)^§^									<0.0001
1	52 (7%)	33 (5%)	43 (5%)	47 (7%)	53 (9%)	28 (10%)	16 (7%)	273 (7%)	
2	109 (14%)	80 (12%)	113 (14%)	70 (11%)	63 (11%)	25 (9%)	29 (12%)	489 (12%)	
3	460 (60%)	402 (58%)	420 (51%)	355 (54%)	280 (47%)	134 (49%)	115 (49%)	2166 (54%)	
4	126 (16%)	121 (17%)	145 (18%)	98 (15%)	80 (14%)	38 (14%)	41 (17%)	649 (16%)	
No data	22 (3%)	59 (8%)	98 (12%)	88 (13%)	114 (19%)	47 (16%)	35 (15%)	452 (11%)	
Median (IQR) CD4 count	136 (51–213)	136 (65–211)	133 (51–212)	124 (33–220)	131 (48–228)	128 (41–251)	149 (57–231)	134 (51–218)	0.23
Vital status									<0.0001
Alive*	503 (65%)	426 (61%)	541 (66%)	467 (71%)	438 (74%)	186 (68%)	187 (79%)	2748 (68%)	
Dead	35 (5%)	40 (6%)	50 (6%)	104 (16%)	63 (11%)	14 (5%)	18 (8%)	324 (8%)	
Lost	231 (30%)	229 (33%)	228 (28%)	87 (13%)	89 (15%)	72 (27%)	31 (13%)	967 (24%)	
Person follow up years	3565.1	2531.8	2158.7	1393.8	936.4	455.2	195.8	11236.8	

* And accessing the clinic, ^§^ % of patients initiating cART.

The characteristics of patients enrolled in the clinic for care, proportion initiating cART, their WHO clinical stages, median CD4 counts and vital status for each calendar year from 2004 to 2010 are shown in Table 2. Overall, the median (IQR) follow-up time on cART for the cohort was 30 (12–54) months. There were 11,236.8 person years of follow up on cART with 2,748 patients (68%) still alive, 967 (24%) lost to follow up and 324 (8%) deaths at the time of closing data for the present analysis.

### Immunological responses/events on cART

#### Changes in CD4 counts with cART

The median (IQR) CD4 count at baseline of 133 (50–218) increased to 314 (204–429) within 6 months of initiation of cART, p<0.0001. This initial increment was sustained at 12 months with a median (IQR) CD4 at 12 months of 355 (244–487), with further increases during follow up to month 90.

#### Immune reconstitution inflammatory disorders

There were 45 documented cases of IRIS with an overall incidence rate of 4.00 (2.92–5.36) events/1000 person years under follow up. Tuberculosis-associated IRIS was the commonest and manifested clinically as new pulmonary infiltrations (n = 16), lymphadenitis (n = 2) and pleural effusions (n = 2) over a median of 2 months (range: 2 to 12 months). Others included herpes zoster (n = 13), cerebral toxoplasmosis (n = 9), CMV retinitis (n = 2) and cryptococcal meningitis (n = 1). The median age of patients experiencing IRIS was 38 years, 33 (73%) were females and the median CD4 count at initiation of cART was 73 (range, 2 to 314) cells/mm^3^. At the close of data for analysis, 27 (60%) patients who developed IRIS were alive, 6 (13%) patients died and 12 (27%) were lost-to-follow up.

#### Incidence and risk factors for immunological failure

Although robust CD4 recovery was noted among most patients on cART, there were 407 immunological failures with a crude event rate of 3.62 (3.28–3.99) per 100 person years. The median (range) time to occurrence of immunological failure on first line cART was 24 (12–78) months. Factors significantly associated with the risk of immunological failure on multivariable Cox proportional hazards analysis were male sex with an adjusted HR of 1.85 (95% CI of 1.47–2.33), initiating cART below 40 years of age with an adjusted HR of 1.25 (1.01–1.55) and baseline CD4 strata below 200 cells/mm^3^ with an adjusted HR of 3.62 (95% CI of 2.59–5.07).

### Clinical events on cART

#### Non-AIDS defining clinical events

There were 41 NADEs with the commonest recorded events being hepatic disorders (n = 20), cardiovascular events (n = 10) and new onset severe renal impairment with eGFR below 30ml/min (n = 6); hepatocellular carcinoma (n = 4) and oesophageal carcinoma (n = 1) were the only non-AIDS defining malignancies. The hepatic disorders noted include liver cirrhosis, hepatitis B transaminitis flares and hepatocellular carcinoma which were all observed in patients with HBsAg sero-positivity. The overall crude NADE incidence rate (95% CI) was 3.64 (2.61–4.95) per 1000 person years as shown in Table 3.

**Table 3:** Frequencies and rates of Non-AIDS defining events (NADEs) and other medical disorders not meeting AIDS-defining or NADEs criteria.

NADEs events	Number of events	Proportion of patients in entire cohort with event (%), n = 4,039	Rate/1000 person years follow up (95% CI)
**Hepatic disorders**			
Chronic liver disease	13	0.3	1.16 (0.62–1.98)
Hepatitis B virus infection flares*	7	0.2	0.62 (0.25–1.28)
**Cardiovascular events**			
Stroke	8	0.2	0.71 (0.31–1.40)
Congestive cardiac failure	2	0.1	0.18 (0.02–0.64)
**Renal disorders**			
Stage 4 or 5 renal impairment	6	0.2	0.53 (0.19–1.16)
**Non-AIDS malignancies**			
Hepatocellular carcinoma	4	0.1	0.36 (0.10–0.91)
Oesophageal carcinoma	1	0.0	0.09 (0.00–0.05)
**Total**	41	1.0	3.64 (2.61–4.95)
**Medical disorders not meeting** **NADEs Non-AIDS defining infectious disorders**			
Malaria	746	18.5	66.39 (61.71–71.32)
Upper and lower respiratory infections	666	16.5	59.27 (54.85–63.95)
Dermatological infections	364	9.0	32.39 (29.15–35.90)
Gastroenteritis	314	7.7	27.94 (24.94–31.21)
Urinary tract infections	151	3.7	13.43 (11.38–15.76)
Ear nose and throat infections	102	2.5	9.08 (7.40–11.02)
Sepsis	29	0.7	2.58 (1.73–3.71)
Bacterial meningitis	5	0.1	0.45 (0.17–1.01)
**Miscellaneous disorders**			
Dyspeptic disorders	73	1.8	6.50 (5.09–8.17)
Post-herpetic neuralgia	52	1.3	4.63 (3.46–6.07)
Seizure disorders	24	0.6	2.14 (1.37–3.18)
Paraparesis (unknown cause)	10	0.3	0.88 (0.43–1.64)
Hemiparesis (unknown cause)	9	0.2	0.80 (0.37–1.52)
Monoparesis (unknown cause)	6	0.1	0.53 (0.19–1.16)
Proximal myopathy	3	0.1	0.27 (0.06–0.78)
**New onset vascular risk factors**			
Hypertension	94	2.3	8.36 (6.76–10.24)
Diabetes mellitus	9	0.2	0.80 (0.37–1.52)

* HBV flare was defined as an elevation of ALT >5X upper limit of normal in a patient with HBSAg sero-positivity.

By far, the commonest medical morbidity (not fulfilling AIDS nor NADEs criteria) was from infectious causes of which 2,377 events were documented (Table 3), with malaria being the commonest event. This is followed briefly by upper/lower respiratory tract infections (n = 666). Of the non-infectious medical comorbidities recorded, 94 patients developed hypertension and 9 Type 2 diabetes mellitus while on cART.

#### Causes and factors associated with AIDS-defining events on cART

Six hundred (600) patients (14.9%) experienced a total of 681 AIDS-defining events during follow up on cART with a crude (95% CI) event rate of 60.60 (56.14–65.33) per 1000 person years of follow up (Table 4). The median (range) time to development of first AIDS-defining event was 6 (2–90) months. Nine patients were documented to have had computed tomographic evidence of an intracranial space occupying lesions, but clinicians did not document the etiology of these mass lesions. For the purposes of the present study, they were judged to be AIDS-defining events. Factors significantly associated with the risk of developing an AIDS-defining event on cART on adjusted analyses were low body mass index below 16 kg/m^2^, WHO clinical stages 3 or 4 at baseline and CD4 strata below 200 cells/mm^3^ at initiation of therapy (Table 5).

**Table 4:** Frequencies and rates of AIDS-defining conditions.

Condition	Frequency of events n (%) n = 681	Proportion of patients in entire cohort with event (%), n = 4,039	Rate/1000 person years follow up (95% CI)
Pulmonary tuberculosis	179 (26.3)	4.4	15.93 (13.68–18.44)
Chronic diarrhoea	155 (22.8)	3.8	13.79 (11.71–16.14)
Esophageal candidiasis	75 (11.0)	1.9	6.67 (5.25–8.36)
Recurrent pneumonia	58 (8.5)	1.4	5.16 (3.92–6.67)
Oral candidiasis	45 (6.6)	1.1	4.00 (2.92–5.36)
Cerebral toxoplasmosis	38 (5.6)	0.9	3.38 (2.39–4.64)
Extrapulmonary tuberculosis	33 (4.8)	0.8	2.94 (2.02–4.12)
Kaposi sarcoma	31 (4.6)	0.8	2.76 (1.87–3.92)
CMV retinitis	17 (2.5)	0.4	1.51 (0.88–2.42)
HIV encephalopathy	16 (2.5)	0.4	1.42 (0.81–2.31)
Cryptococcal meningitis	12 (1.8)	0.3	1.07 (0.55–1.87)
Intracranial space occupying lesion*	9 (1.3)	0.2	0.80 (0.37–1.52)
Non-Hodgkin's disease	5 (0.7)	0.1	0.44 (0.14–1.04)
HIV wasting syndrome	3 (0.4)	0.1	0.27 (0.05–0.78)
*Pneumocystis jirovercii* pneumonia	2 (0.3)	0.0	0.18 (0.02–0.64)
CNS lymphoma	1 (0.1)	0.0	0.09 (0.00–0.50)
Herpes esophagitis	1 (0.1)	0.0	0.09 (0.00–0.50)
Invasive cervical carcinoma	1 (0.1)	0.0	0.09 (0.00–0.50)
**Total**	**681**		**60.60 (56.14–65.33)**

AIDS defining events were defined using WHO clinical criteria. * Causes of Intracranial space occupying lesion on CT scan were not documented but were presumed to be AIDS-defining events.

**Table 5:** Univariate and multivariate analysis of factors associated with risk of developing AIDS on cART.

Variable	Person follow-up time (yrs)	Number of events	Crude event rate (/1000 py), 95% CI	Unadjusted HR (95% CI)	p-value	Adjusted HR (95% CI)	p-value
**Sex**							
Male	3467	193	55.67 (48.09–64.10)	1.06 (0.89–1.25)	0.53	–	–
Female	7770	406	52.25 (47.29–57.59)	1.00			
**Age**							
**≥**40 years	5161	268	51.93 (45.90–58.53)	1.00 (0.94–1.07)	0.90	–	–
<40 years	6082	331	54.42 (48.72–60.61)	1.00			
**WHO stage**							
3 or 4	7803	477	61.13 (55.77–66.87)	1.73 (1.37–2.18)	<0.0001	1.45 (1.13–1.86)	0.0031
1 or 2	2370	84	35.44 (28.27–43.88)	1.00		1.00	
**Baseline CD4 strata**							
<200	7706	481	62.42 (56.96–68.25)	2.00 (1.64–2.45)	<0.0001	1.87 (1.49–2.35)	<0.0001
>200	3462	117	33.80 (27.95–40.50)	1.00		1.00	
**Baseline BMI**							
<16 kg/m^2^	901	99	109.9 (89.3–133.8)	1.91 (1.54–2.38)	<0.0001	1.53 (1.20–1.94)	0.0005
**≥**16 kg/m^2^	9189	483	52.56 (47.98–57.47)	1.00		1.00	
**Baseline Hb**							
<8g/dl	1176	87	73.98 (59.25–91.25)	0.92 (0.83–1.01)	0.06	1.01 (0.78–1.31)	0.94
**≥**8g/dl	8590	494	57.51 (52.55–62.81)	1.00		1.00	
**NRTI backbone**							
D4T plus 3TC	5276	322	61.03 (54.55–68.07)	1.18 (1.01–1.39)	0.04	1.08 (0.90–1.30)	0.09
AZT plus 3TC	5940	274	46.13 (40.83–51.93)	1.00		1.00	
**NNRTI**							
Efavirenz	6120	330	53.92 (48.26–60.06)	0.92 (0.78–1.08)	0.29	–	–
Nevirapine	5013	261	52.06 (45.94–58.78)	1.00			
**Adherence**							
Poor	3835	237	61.80 (54.18–70.18)	1.17 (0.99–1.38)	0.06	1.06 (0.89–1.27)	0.51
Excellent	6345	363	57.21 (51.48–63.41)	1.00		1.00	

Of the 600 patients who developed AIDS-defining events on cART, 317 (52.8%) were alive as at time of closure of data but 153 (25.5%) were subsequently lost-to-follow up and 130 (21.7%) died of AIDS. The median (range) duration between the first AIDS-defining diagnosis and death was 0.25 months (0 to 72 months) and that for loss-to-follow up was 4 months (0 to 76 months). Patients who developed an AIDS-defining event and were alive at the time of closure of data for analysis survived for a median of 34 months (range of 0 to 88 months).

### The incidence, causes and risk factors associated with mortality

There were 324 (8.0%) deaths over the 11,263.8 person follow-up years giving a crude (95% CI) event rate of 28.83 (25.78–32.15) deaths per 1000-person follow up years. The median (range) time to death was 2 months (0–66) by Mann Whitney's U-test. 202 (62.3%) deaths occurred within the first 90-days of initiation of therapy, 88 (27.2%) from month 4 to month 12, 16 (4.9%) within the second year, 9 (2.8%) within the third year, 3 (0.9%) within the fourth year and 6 (1.9%) within the fifth year of follow up. The Kaplan Meier estimated cumulative incidence of mortality at 6, 12, 36, and 72 months were 6.8%, 7.9%, 9.0% and 9.8% respectively and of attrition (including 324 deaths and 958 loss-to-follow ups) from the programme were 17.3%, 20.7%, 27.8%, 34.5% and 36.5% respectively.

As shown in Table 6, significant factors associated with mortality in Cox proportional hazards model were male gender with an adjusted HR (95% CI) of 1.69 (1.29–2.21), advanced HIV disease – Stages 3 or 4 – with an adjusted HR (95% CI) of 2.20 (1.42–3.41), low CD4 strata below 200 cells/mm^3^ with an adjusted HR (95% CI) of 2.39 (1.64–3.49), baseline BMI below 16 kg/m^2^ with an adjusted HR (95% CI) of 2.60 (1.92–3.53) and starting on an NRTI backbone of d4T plus 3TC with an adjusted HR of 1.60 (1.21–2.11).

**Table 6:** Univariate and multivariate analysis of factors associated with death on cART.

Variable	Patient follow-up time (years)	Number of events	Crude event rate (/1000 person years)	Unadjusted HR (95% CI)	p-value	Adjusted hazard ratio (95% CI)	p-value
**Sex**							
Male	3467	188	54.23 (46.75–62.56)	1.71 (1.37–2.14)	<0.0001	1.69 (1.29–2.21)	0.0001
Female	7770	136	17.50 (14.69–20.70)	1.00		1.00	
**Age**							
**≥**40 years	5161	143	27.71 (23.35–32.64)	0.94 (0.75–1.17)	0.55	–	–
<40 years	6082	180	29.60 (25.43–34.25)	1.00			
**WHO stage**							
3 or 4	7803	277	35.50 (31.44–39.94)	3.52 (2.34–5.30)	<0.0001	2.20 (1.42–3.41)	0.0004
1 or 2	2370	25	10.55 (6.82–15.57)	1.00		1.00	
**Baseline CD4 strata**							
<200	7706	276	35.81 (31.72–40.30)	3.13 (2.29–4.29)	<0.0001	2.39 (1.64–3.49)	<0.0001
>200	3462	45	13.00 (9.48–17.39)	1.00		1.00	
**Baseline BMI**							
<16 kg/m^2^	901	88	97.67 (78.33–120.33)	3.79 (2.95–4.85)	<0.0001	2.60 (1.92–3.53)	<0.0001
**≥**16 kg/m^2^	9189	214	23.29 (20.27–26.63)	1.00		1.00	
**Baseline Hb**							
<8g/dl	1176	76	64.63 (50.92–80.89)	2.43 (1.88–3.16)	<0.0001	1.28 (0.95–1.75)	0.11
**≥**8g/dl	8590	227	26.43 (23.10–30.10)	1.00		1.00	
**NRTI backbone**							
D4T plus 3TC	5276	216	40.94 (35.66–46.78)	2.04 (1.62–2.58)	<0.0001	1.60 (1.21–2.11)	0.001
AZT plus 3TC	5940	106	17.85 (14.61–21.58)	1.00		1.00	
**NNRTI**							
Efavirenz	6120	212	34.64 (30.13–39.63)	1.43 (1.14–1.81)	0.0024	1.15 (0.87–1.51)	0.32
Nevirapine	5013	108	21.54 (17.67–26.01)	1.00		1.00	
**Adherence**							
Poor	3835	147	38.33 (32.39–45.05)	1.52 (1.22–1.89)	0.0002	1.21 (0.95–1.56)	0.12
Excellent	6345	177	27.90 (23.94–32.32)	1.00		1.00	

In a series of 188 hospital-confirmed deaths, the causes of death are as shown in Table 7. The leading causes of death in this subset of patients were pulmonary tuberculosis, severe diarrhoea with hypovolaemic shock, severe anaemia, pneumonia and sepsis.

**Table 7:** Causes of death among 188 patients who died in hospital while on first line cART.

Cause of death	Frequency
Pulmonary tuberculosis	37
Gastroenteritis with hypovolemic shock	30
Severe anemia	19
Pneumonia	18
Sepsis	16
Cerebral toxoplasmosis	12
HIV Encephalopathy	7
TB Immune reconstitution inflammatory syndrome	7
Disseminated Kaposi sarcoma	6
Enteric fever	4
End stage kidney failure	4
Lactic acidosis	4
Bacterial meningitis	4
Chronic liver disease	2
Cryptococcal meningitis	2
Steven's Johnsons syndrome due to nevirapine	2
Miscellaneous*	14
TOTAL	188

* Miscellaneous comprises of 1 case each of acute abdomen, amoebic liver abscess, CNS lymphoma, gluteal abscess, hepatocellular carcinoma, HBV flare, high grade non-Hodgkin's lymphoma, hyperglycemic hyperosmolar syndrome, strangulated umbilical hernia, otitis media, *Pneumocystis jirovercii* pneumonia, systemic candidiasis, tuberculous colitis, fulminant vasculitis with gangrene of toes and fingers.

#### Treatment-limiting toxicities

Overall 35.3% (n = 4,039) of patients experienced toxicity during follow up. There were 1,603 documented episodes of cART-related toxicities among patients who started first line cART with an event rate of 14.3 events/100 person-years (95% CI, 13.6 to 15.0/100 person-years). As shown in Table 8, severe anemia, skin rash and neuropsychiatric toxicity were the commonest reported adverse events. 271 patients representing 6.8% of the entire cohort switched therapy due to toxicity.

**Table 8:** Frequencies of specific toxicities and treatment switches among Ghanaian cohort on long-term cART.

Toxicity	Frequency of toxicity among patients who developed any toxicity on ART n (%), n = 1,603	Proportion of patients who developed toxicity n (%)§ n = 4,039	Frequency of treatment switches due to specific toxicity# n (%)	Proportion of patients with treatment switch due to toxicity, (%) n = 4,039
Anemia	675 (42.1)	527 (13.0)	62 (11.8)	1.5
Skin rash	295 (18.4)	281 (7.0)	44 (15.7)	1.1
Neuropsychiatric toxicity	235 (14.7)	218 (5.4)	39 (17.9)	1.1
Peripheral neuropathy	181 (11.3)	181 (4.5)	83 (45.9)	2.1
Severe hepatotoxicity	143 (8.9)	143 (3.5)	8 (5.6)	0.2
Lipoatrophy	40 (2.5)	40 (1.0)	34 (85.0)	0.8
Ptylism	14 (0.9)	14 (0.3)	0 (0.0)	0.0
Gastrointestinal disorders	12 (0.7)	12 (0.3)	0 (0.0)	0.0
Lactic acidosis	4 (0.2)	4 (0.1)	0 (0.0)	0.0
Myalgia	3 (0.2)	3 (0.1)	0 (0.0)	0.0
Hyperpigmentation	3 (0.2)	3 (0.1)	0 (0.0)	0.0
Pancreatitis	1 (0.1)	1 (0.0)	1 (100.0)	0.0
Column total	1,603	1,427 (35.3)	271	6.8

# This refers to the number of patients switching treatment due to specific toxicity specified on the row. % was determined by dividing the number of patients switching treatment due to a specific toxicity by the total number of patients with that particular toxicity in question.

§ n(%) n refers to the number of patients who experienced specific toxicity and the % refers to number of patients with toxicity divided by the total number of patients starting ART which was 4,039. An individual patient may experience more than one toxicity during follow up and may experience a specific toxicity more than one episode during follow up.

#### Rates of major clinical events on cART

**Figure 1:**
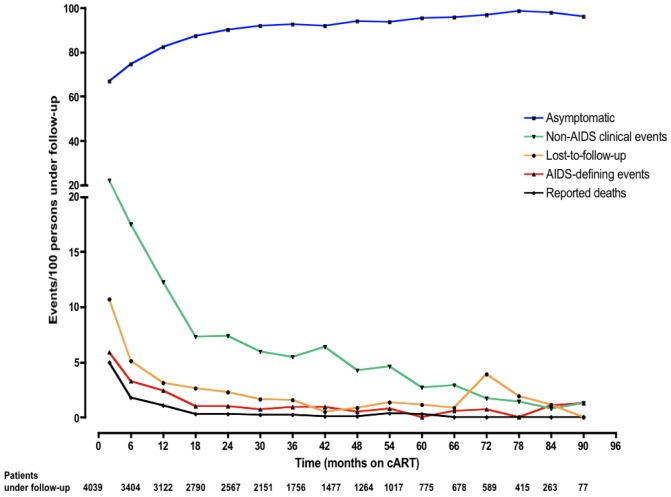
Six monthly incidence rates of Non-AIDS and AIDS events, deaths, loss to follow up and asymptomatic events among Ghanaian HIV-infected patients on long-term cART. Non-AIDS events comprised all medical conditions which are non-AIDS defining by WHO criteria.

Figure 1 depicts the overall decline in the rates of AIDS-defining events, non-AIDS clinical events, mortality and loss to follow up over time on cART. The highest incidence rates of these events were observed within the first two months of initiating cART with rates of 22.1 non-AIDS clinical events per 100 patients, 10.7 lost-to-follow up per 100 patients, 5.9 AIDS defining events per 100 patients and 5.0 deaths per 100 patients under follow-up. One hundred and thirty-five (135) patients (3.3%) starting NNRTI-based first line cART were subsequently switched to a PI-based second line therapy on account of treatment failure at closure of data for analysis.

## Discussion

This study is among the largest cohort studies documenting morbidity and mortality in an ART programme in sub-Saharan Africa. The data presented in this paper show that there is a sustained and robust immunological recovery on cART among this cohort of HIV-infected patients. This immune restoration accompanied improvements in morbidity as evidenced the steady decline in the incidence of deaths, losses to follow-up, AIDS-defining and non-AIDS defining clinical events. Attrition from the programme, due mainly to deaths and loss-to-follow up as well as AIDS-defining events, occurred predominantly within the first year of treatment and were predicted by clinical and laboratory indicators of advanced HIV disease at initiation of cART.

Several studies from both developed [19]–[23] and developing countries [1], [24]–[29] have reported short-, medium- and recently long-term CD4 responses on cART. The findings from this study show that within a programme setting in sub-Saharan Africa, immunological responses to cART are sustainable over the long-term on a limited repertoire of first-line therapy. As expected, the median (IQR) CD4 counts at initiation of therapy of 133 (50–218) cells/mm^3^ were low and comparable with those from several cohorts from developing countries [24]–[34] and serve as an indicator of the advanced stages of immunosuppression at which cART is initiated in these settings [35]. This notwithstanding, cART was associated with a near doubling in median CD4 counts within 6 months of therapy followed by more gradual and sustained increases throughout the period of observation for patients remaining under care. The vigorous restitution of CD4 cell counts within the first six months of therapy was accompanied by the occurrence of IRIS in some patients, of which tuberculosis was the commonest reported cause followed by herpes zoster and cerebral toxoplasmosis. Admittedly, compared with other cohorts where the reported frequency of IRIS has varied between 10% to 25% of all patients initiating cART [18], [36]–[40], the overall frequency of 1.1% of IRIS reported among this cohort is low, and probably reflects the difficulties in the diagnosis of this clinical syndrome within settings of limited diagnostic support. Events were considered as IRIS when clinicians felt they had sufficient clinical and laboratory evidence to support the diagnosis.

For patients who stayed on cART for more than a year, 407 (10.1%) developed immunological failure according to WHO guidelines but only 135 (3.3%) patients in the cohort were switched to second line therapy. The spectra of clinical events classified as IRIS overlaps closely with those diagnosed as AIDS-defining events which occurred at a frequency of 17%. AIDS-defining events on cART occurred at a median time of 6 months, were predicted by low CD4 cell counts, prior AIDS and low BMI at initiation of therapy and were associated with an increased risk of subsequent attrition from the programme. These show that the occurrence of AIDS-defining events in the proximal phases of cART is an indicator of early treatment failure and is representative of progression of HIV disease which cART may be incapable of halting, even when appropriate treatment for opportunistic infections is concurrently commenced. The implication is that cART should be started much earlier, requiring patients to be diagnosed earlier, as has been suggested by others [35], [41].

The crude incidence rates of Non-AIDS-defining events of 3.64/1000 person-years is lower compared to that of cohorts from Botswana [13] and the United States [13] of 10.0 py, and 12.4 py, respectively, that of a multicentre Latin American cohort [42] of 8.4/1000 person-years and that of the Eurosida cohort [11] of 17.7 (95% CI of 16.6 to 18.7/1000 py, n = 12,844). The median follow up for the cohorts from Botswana [13], the USA [13], and Latin America [42] were 36 months, 18 months and 30 months respectively which is comparable with the 30 months of follow up of this cohort. However, the retrospective nature of the present study and the relatively short median follow-up in this cohort could have accounted for the lower incidence rates of NADEs. Of the 41 NADEs in this cohort, hepatic disorders (n = 20) were the commonest NADE followed by cardiovascular events (n = 10), end-stage renal disease (n = 6) and non-AIDS malignancies of which hepatocellular carcinoma (n = 4) and one case of oesophageal carcinoma were reported. Overall, 24 of the 41 documented NADEs were associated with HBSAg seropositivity making HBV co-infection an important determinant of NADEs in this cohort. The order of events were different from those in the cohort from Botswana where out of 18 NADEs, 9 were cardiovascular, 4 were renal, five were malignancies and none were of hepatic in etiology [13]. As a composite outcome measure, non-AIDS events are a heterogeneous collection of several end-points with multifactorial aetiologies and risk factors, and also dependent on the presence of chronic hepatitis B co-infection and chronic vascular inflammation due to persistence of HIV viral replication [43]–[52]. The multi-dimensional composition of risk factors for NADEs together with the relatively limited observation of events and some missing data restricted analysis of risk factors for these events in this cohort.

Malaria was the leading cause of non-AIDS-defining infectious morbidity on cART and was often treated with artemisinin-based antimalarial therapy. The fact that within this HIV cohort, tuberculosis and malaria were the leading causes of AIDS and non-AIDS infectious morbidity respectively underscores their importance among the most important public health challenges Sub-Saharan Africa is attempting to overcome. Unlike in developed countries where AIDS-related mortality on cART is being superseded by Non-AIDS-related mortality [52], causes of death in this cohort were predominantly driven by AIDS-related events of which tuberculosis was the leading cause followed by gastroenteritis with hypovolemic shock, severe anaemia and pneumonia, as in other cohorts from developing countries. Of the NADEs, end-stage kidney disease and liver-related disease were among the common causes of mortality. It is also noteworthy that 6 deaths were attributed directly to cART-related toxicity: two cases of Stevens Johnson's syndrome from nevirapine and four cases of clinically suspected lactic acidosis from stavudine. cART toxicity is a cardinal factor influencing the durability and therefore the effectiveness of cART and 35.3% of patients experienced one form of toxicity or the other with 6.8% discontinuing therapy on account of toxicity. Use of a stavudine was independently associated with increased risk of death, hence the withdrawal of this antiretroviral from the Ghanaian ART programme is justified. Given the high prevalence of renal impairment among this cohort (38.8%) [53], replacement of stavudine by tenofovir should however be approached with caution due to the well-known association between tenofovir and risk for renal tubular toxicity. It is encouraging that the DART study has provided some reassuring data on the safety of tenofovir among Africans [54]. Furthermore given the high prevalence of hepatitis B co-infection in this cohort, the benefits of tenofovir in slowing the progression of liver disease may outweigh possible renal toxicity.

The analyses presented in this study have limitations worth noting. As over 60% of patients presenting to the clinic did not start cART, and many with advanced HIV/AIDS were lost to follow-up, it is likely there were many deaths in this group. Hence the population studied was skewed towards those patients who survived long enough to start cART, who may have had different causes of death to those not starting cART. The trajectories of CD4 over time reflect those of patients who remained on cART and thus are influenced by survivor bias. Furthermore the sources of ascertainment of causes of deaths in a significant proportion of patients were by verbal autopsy by relatives whose accuracy may be limited. In spite of these the specific causes of death could be verified by medical certification in 188 (58%) out of 324 events. Similarly, the classification of AIDS-defining events, IRIS and NADEs were influenced by availability of data and arrived at by consensus between the authors and local experts.

In summary, cART is effective over the long-term for a substantial proportion of this Ghanaian cohort of HIV-infected patients. Within the constraints of limited resources as it pertains to the ART programme in Ghana, cART was associated with a sustained and durable immunological recovery in most patients, an improvement in morbidity from HIV-infection and trend towards reduction in mortality over the long-term among patients remaining on therapy and in care.
